# CDMSP: a convolutional dense multi-scale pooling framework for low-light colonoscopy image enhancement

**DOI:** 10.3389/frai.2026.1903125

**Published:** 2026-08-26

**Authors:** Mohan K., Gopinath Palanisamy, Nisha J S.

**Affiliations:** School of Electronics Engineering, Vellore Institute of Technology, Vellore, Tamil Nadu, India

**Keywords:** colonoscopy, colorectal cancer, computer-aided diagnosis, deep learning, low-light image enhancement, multi-scale feature extraction

## Abstract

Colorectal cancer (CRC) remains one of the main cancer-related causes of death worldwide, and colonoscopy is an effective method for early identification of CRC and substantially reducing the risk. Colonoscopy images are often affected by non-uniform illumination and low-light conditions, which make it difficult to visualize mucosal textures and lesion margins. In this study, we introduce a low-light colonoscopy image enhancement framework, Convolutional Dense Attention Network (CDAN)-Dense- Multi-Scale Pooling (MSP) (CDMSP), to address this problem. The framework centers around a CDAN, which is constructed through dense connections and attention mechanisms to increase feature propagation and emphasize diagnostically important areas while reducing noise and lighting artifacts. In addition, a small MSP module is used to capture contextual information at different spatial scales, thereby helping preserve the details of the structures and edges. The overall loss function, which is a combination of structural similarity, perceptual similarity, edge-based contrast, and color fidelity, is used to maintain a balance between contrast enhancement and the preservation of anatomy. According to the experimental results, the proposed method achieved a Structural Similarity Index Measure (SSIM) of 0.8757, a Learned Perceptual Image Patch Similarity (LPIPS) of 0.0742, an edge-based contrast measure (EBCM) of 0.5601, and a Color Fidelity Index (CFI) of 95.1760. It is extremely efficient with an inference time of [23.3 milliseconds (ms), 42.99 frames per second (FPS)], making it suitable for real-time clinical use.

## 1 Introduction

**Figure 1:**
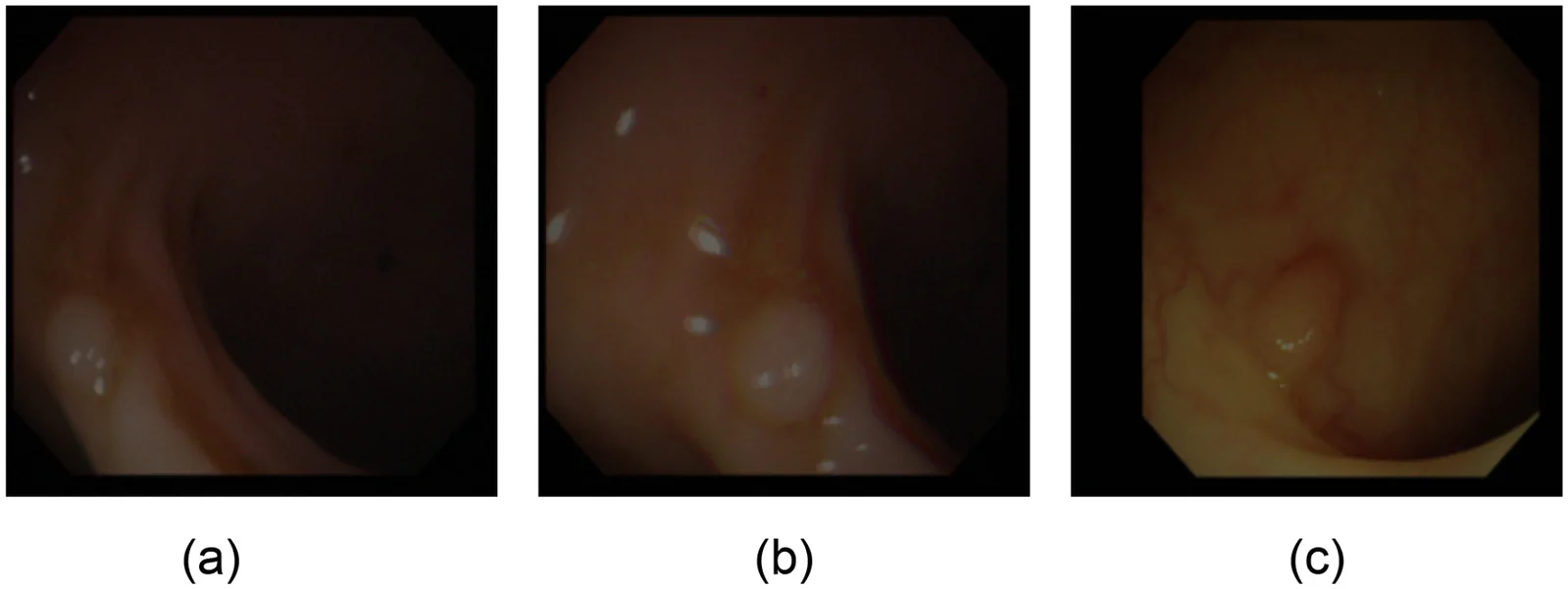
Representative low-light colonoscopy scenarios encountered in clinical practice: **(a)** underexposed image, **(b)** uneven illumination with poor contrast around a polyp, and **(c)** faded appearance with reduced mucosal detail visibility.

CRC is the third most common cancer diagnosis and the second leading cause of cancer-related deaths worldwide (Sung et al., 2021). Globally, CRC accounts for approximately 10.2% of cancer cases and 9.2% of cancer-related deaths. It is the third most common cancer, following lung and breast cancers (Bray et al., 2018). Colonoscopy is the primary screening method for the early detection of CRC. However, it still faces challenges, such as insufficient contrast visibility and difficulties in recognizing small or flat lesions. Studies have shown that the average Adenoma Miss Rate (AMR) is approximately 24%, with the rate rising to 27% for small adenomas [ ≤ 5*millimeter*(*mm*)] that have only been detected by subsequent colonoscopy procedures (Rex et al., 1997). Research suggests that the AMR could be as high as 26%, indicating that several precancerous lesions might be ignored during standard colonoscopy procedures (Zhao et al., 2019). Advanced imaging techniques, such as narrow-band imaging (NBI) and Linked Color Imaging (LCI), increase lesion visibility by enhancing color contrast and brightness. However, problems such as dimly lit situations, dark patches, and low contrast can still make it difficult to identify lesions (Tanaka et al., 2019). Figure 1 shows common features in images and videos, such as very dark areas, light that is on and off, and low contrast, especially near polyps. These problems make it very difficult for the human eye to notice and identify tissue features. These limitations can prevent the accurate detection and understanding of findings during colonoscopy.

Yue et al. (2023) presented a Deep Pyramid Enhancement Network (DPENet) that can be used to improve low-light endoscopic images by extracting multi-scale features from them. The pyramid architecture not only considers global and local information but also uses it to correct the lighting, remove noise, and produce clearer structural details for a reliable analysis.

The main contributions of this study are summarized as follows:

- The proposed CDMSP network integrates the CDAN architecture with dense blocks and MSP to improve the gradient flow and feature propagation and capture the representation of key diagnostic areas in colonoscopy images.
- The CDAN is essentially a Convolutional Block Attention Module (CBAM) that is applied to convolutional feature maps. Placing CDAN blocks in sequence after dense layers enables the network to emphasize key diagnostic features and reduce noise and artifacts.
- The MSP module merges features from different spatial scales using a 1 × 1 bottleneck. This widening of the receptive field not only brings the entire global context at one glance but also makes the model more robust to challenges caused by changes in illumination, texture differences, and complex structures.
- The model utilizes residual learning, where the desired improvement is added to the initial image, ensuring that the final result remains in the range [0, 1]. This residual approach not only makes the training process more stable and converges faster but also effectively prevents over-enhancement in medical images.
- The objective of the network is to minimize the hybrid loss, which consists of the SSIM, LPIPS, EBCM, and CFI. These losses guide the model to enhance the contrast and visibility without compromising the anatomical details and the naturalness of the colors.

The proposed method improves the quality of low-light colonoscopy images by employing a dataset of 2,000 images for training and evaluation. However, extensive validation with larger and more heterogeneous clinical datasets and real-time trials is necessary for reliable clinical implementation. The remainder of this paper is organized as follows: Section II deals with enhancing low-light image techniques and deep learning methods for feature extraction, while Section III illustrates the proposed methodology. Section IV describes the experimental setup, Section V discusses the analysis of the results, and Section VI concludes this paper.

## 2 Literature review

### 2.1 Low-light image enhancement techniques

Traditional low-light enhancement methods, such as histogram equalization and Retinex-based techniques, rely on simple computations; therefore, these methods are widely used. However, these methods typically produce visual artifacts. For example, noise amplification, color distortion, and halo effects restrict their success in very low-light conditions (Jiang et al., 2025). Wei et al. (2018) developed an innovative deep learning system aimed at mimicking the Retinex theory, which separates the input images into reflectance and illumination images via a Decomposition Network (DecomNet), and an Enhancement Network (EnhanceNet) is used for illumination enhancement. In addition, the technique denoises the reflectance layer and demonstrates superior visual quality and structure preservation on the low-light (LOL) dataset compared with conventional methods.

Gómez et al. (2019) introduced a modified U-Net, a U-shaped convolutional Network to improve low-light laryngeal films, employing Perlin noise to create synthetic dark examples for training purposes. Their approach enhances the brightness, contrast, and visual clarity relative to conventional methods, mitigating problems such as color distortion and feature degradation in low-light images. Guo et al. (2020) developed a deep learning approach called zero-reference deep curve estimation (Zero-DCE), which brightens low-light images by determining pixel-wise curves for changing illumination without requiring paired training data. It employs specially designed non-reference loss functions to guide the enhancement, producing visually realistic results using a simple and efficient model.

Spatial domain illumination adjustment and Wavelet Attention Network (SWANet), a method proposed by He et al. (2023), is a dual-phase framework for low-light image enhancement that combines a multi-scale illumination adjustment network (MIANet) for multi-scale illumination enhancement and a Wavelet-based Noise Enhancement Network (WNENet) for wavelet-based denoising and detail restoration. The experimental results confirm visually clearer images with the same structural features in low-light conditions. RetinexFormer was introduced by Cai et al. (2023), and it is a transformer-based architecture that reinterprets Retinex theory in a single unified model with illumination-guided attention. This approach not only enhances the lighting but also recovers lost details. It outperforms previous Convolutional Neural Network (CNN) -based methods for enhancing low-light images.

Lightweight Temporal Diffusion (LighTDiff) was introduced by Chen et al. (2024) as a lightweight diffusion framework for enhancing low-light surgical endoscopic images. The Temporal Light Unit (TLU) and Chroma Balancer (CB) components are used to maintain consistent denoising and color fidelity while achieving better quality at a lower computational cost. Dong et al. (2025) proposed a technique Self-Guided Low-Light Image Enhancement (SGLLIE) to enhance low-light images by embedding stable structural features in the CNN—Transformer framework via a cross- attention mechanism. The proposed method not only improves the results under extremely dark conditions but also preserves the structures and the real quality of the images on standard datasets.

### 2.2 Feature extraction

AlexNet, presented by Krizhevsky et al. (2012), attained exceptional performance on the ImageNet Large-Scale Visual Recognition Challenge (ILSVRC) using Graphics Processing Unit (GPU) acceleration, Rectified Linear Unit (ReLU) activation, data augmentation, and dropout techniques. This study illustrates the efficacy of deep learning for large-scale visual identification and represents a significant milestone in contemporary computer vision. OverFeat, presented by Sermanet et al. (2013), is a convolutional network capable of simultaneously performing image categorization, localization and object detection. Using multi-scale sliding windows and bounding box regression on the ImageNet dataset, experiments demonstrated that a single deep architecture could effectively handle several visual recognition tasks simultaneously.

Simonyan and Zisserman (2014) proposed Visual Geometry Group (VGG) Network, where they discovered that stacking multiple 3 × 3 convolutional layers significantly improves the image recognition performance. VGG, which was trained on ImageNet, achieved a top-5 error rate of 7.3% (6.8% with ensembling) and was extremely transferable to different vision problems. GoogLeNet, proposed by Szegedy et al. (2015), introduced an inception framework. This approach captures features at multiple scales by employing filters of different sizes while maintaining computational efficiency. The model not only used significantly fewer parameters than earlier deep networks but also excelled in the 2014 ILSVRC competition.

Residual Networks (ResNet) proposed by He et al. (2016) resolved the problem of degradation in very deep networks by using identity shortcut connections. These residual connections help propagate gradients smoothly and consistently, which in turn enables effective training of very deep models, resulting in better performance on benchmark datasets, such as ImageNet and Common Objects in Context (COCO). SqueezeNet was created by Iandola et al. (2016) and achieved results similar to AlexNet with approximately 50 times fewer parameters using fire modules and deferred downsampling. The design appears to maintain a high performance without requiring many calculations.

In a Dense Convolutional Network (DenseNet), all layers are directly connected to each other from the first to the last. Such a network not only improves the flow of features from one layer to another, but also encourages the reuse of features and reduces the number of parameters. This was the main idea behind the architecture introduced by Huang et al. (2017). U-Net Ronneberger et al. (2015) follows a shrinking path to gather context and a growing path to determine locations through the connections between the encoder and decoder.

## 3 Methodology

**Figure 2:**
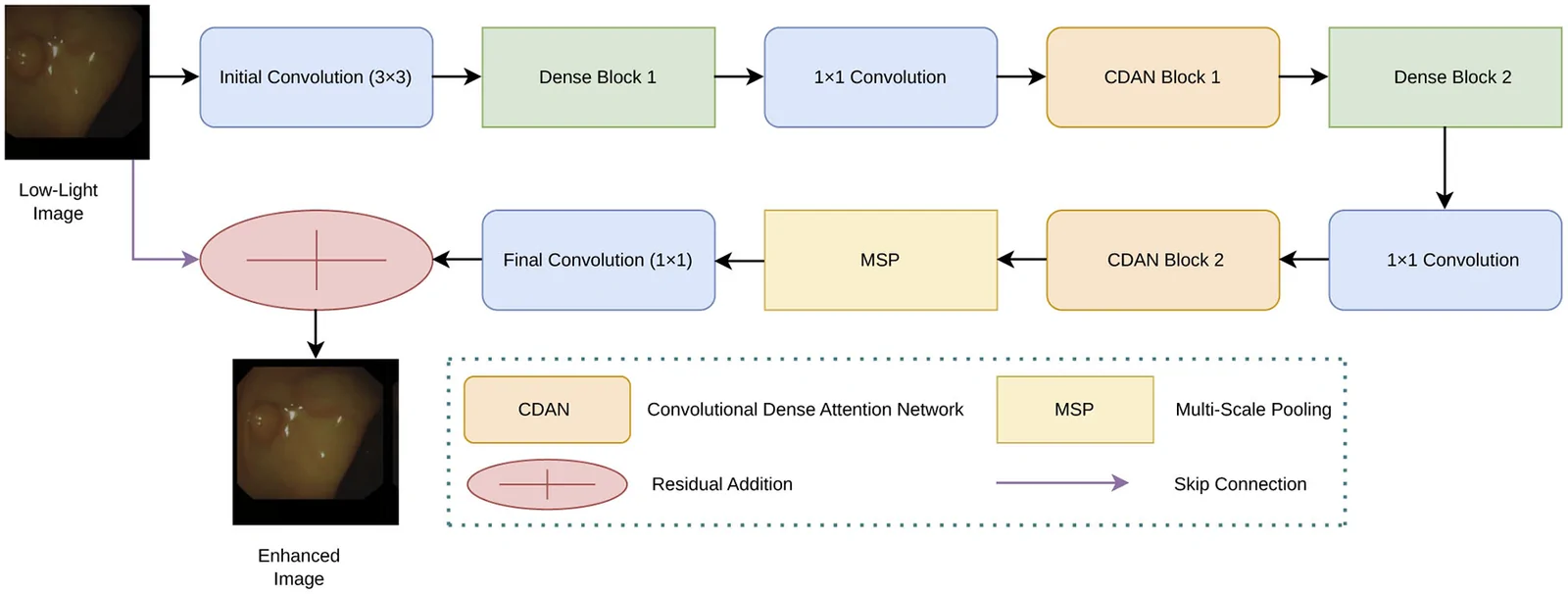
Overall architecture of the proposed CDMSP network.

Colonoscopy images captured in low light often have poor lighting, low contrast, and noise, which obscure important structures and make polyp diagnosis difficult. The CDMSP deep network was designed to generate visually clearer images by highlighting the mucosal textures, edges, and detailed structures while simultaneously reducing light noise and artifacts (Figure 2). The model consists of five major parts: the initial convolution, dense block, CDAN block, MSP, and residual output block.

The basic layer extracts the initial features, whereas the dense and CDAN incorporate CBAM-based blocks to progressively refine and expand the features through dense connectivity and attention mechanisms. The MSP module captures multi-scale contextual information for better representation of structural patterns. Simultaneously, the residual output block generates a restored image that is enhanced and agrees with the restoration. The proposed CDMSP framework comprises dense feature reuse, attention improvement, and multiscale context aggregation. These components work together to brighten low-light colonoscopy images without compromising the diagnostic-relevant information.

### 3.1 Initial convolution (3x3)

The initial step of the proposed model is a 3 × 3 convolutional layer that transforms the input Red, Green, Blue (RGB) colonoscopy image into a higher-dimensional feature space. Initially, convolution is performed with 64 feature channels using a 3 × 3 kernel, one-pixel zero-padding, and a stride of 1 to maintain the original image size during the feature extraction process. Unless stated otherwise, all subsequent 3 × 3 convolutional layers also used one-pixel padding to preserve the resolution of the feature maps. The small kernel efficiently gathers local information and simultaneously maintains detailed spatial information; therefore, it portrays each pixel as a vector of multichannel features. Such a portrayal is especially necessary for the correct retention of highly sensitive structures, such as polyps, mucosal textures, and vascular patterns, under low-light conditions. The extracted features were then forwarded to the enhancement modules for further refinement and reconstruction, resulting in improved visibility and contrast.

### 3.2 Dense block

To improve feature propagation and alleviate the problem of vanishing gradients, Huang et al. (2017) introduced a dense-block structure. This design uses dense connections, meaning that every layer receives feature maps not only from its immediate previous layer but also from all layers before it. Thus, the network efficiently reuses the features, and the gradient flow is boosted. The output of the *l*-th layer in the dense block is computed as shown in Equation 1.


$$\begin{array}{l} F_{l}=\sigma \left(W_{l}*\left[F_{0},F_{1},\ldots ,F_{l-1}\right]\right) \end{array}$$ (1)

Where *F*_*l*_ is the output feature map of the *l*-th layer, σ(·) denotes the ReLU activation function, *W*_*l*_ represents the convolutional weights, * represents convolution, and [*F*_0_, *F*_1_, …, *F*_*l*−1_] depicts the concatenation of the previous feature maps. In the proposed CDMSP model, each dense block is composed of three densely connected convolutional layers with a growth rate of eight. After the features are concatenated, a 1 × 1 convolution is utilized to reduce the concatenated feature maps back to 64 channels before they proceed to the subsequent CDAN block. Dense blocks allow the reuse of features, which not only protects the texture and color information but also enhances low-light colonoscopy images, preserving the anatomical details. The enhanced feature maps are then passed on to the next layers for further refinement and reconstruction, subsequently improving the visibility of faint structures, such as polyps, in the final upgraded images.

### 3.3 Convolutional block attention module (CBAM)

Woo et al. (2018) introduced the CBAM, that is a light and easy-to-use attention mechanism designed for CNN feature maps in which channel and spatial attention are applied one after the other to enhance feature representation. The channel attention module captures inter-channel dependencies to emphasize the most characteristic elements, such as edges, textures, and lesions. In the proposed CDMSP model, the CBAM module employs a channel attention mechanism with a reduction ratio of 16, followed by a spatial attention module that employs a 7 × 7 convolution to enhance the feature representation.

#### 3.3.1 Channel attention

The channel attention mechanism identifies the most effective channels in the feature map, enabling the network to focus on features relevant for diagnosis, such as edge textures and lesion areas. The channel attention map is derived using (Equation 2).


$$\begin{array}{l} {\text{M}}_{c}=\sigma \left(\text{MLP}\left(\text{AvgPool}\left(\text{F}\right)\right)+\text{MLP}\left(\text{MaxPool}\left(\text{F}\right)\right)\right) \end{array}$$ (2)

where **F**∈ℝ^*C*×*H*×*W*^ is an input feature map. AvgPool(·) and MaxPool(·) functions generate channel descriptors by globally pooling the spatial dimensions. The descriptors are first processed by a Multilayer Perceptron (MLP) and then by a sigmoid activation function σ(·) to produce the channel attention map **M**_*c*_, which assigns importance weights to each channel.

The channel-refined feature map is derived using (Equation 3).


$$\begin{array}{l} {\text{F}}^{\prime }={\text{M}}_{c}⊙\text{F} \end{array}$$ (3)

where ⊙ represents element-wise multiplication, **M**_*c*_ is the channel attention map, and **F**′ is the feature map after channel attention refinement.

#### 3.3.2 Spatial attention

The spatial attention mechanism identifies important regions in the feature map, enabling the network to focus on the key spatial locations. It highlights polyp margins and mucosal patterns, for example, in low-light colonoscopy images, to reveal fine structures that may be linked to the disease and lay the groundwork for a more accurate diagnosis. The spatial attention map is given by Equation 4.


$$\begin{array}{l} \text{M}s=\sigma \left(\text{Conv}7\times 7\left(\left[\text{Av}{\text{g}}_{c}\left({\text{F}}^{\prime }\right),\text{Ma}{\text{x}}_{c}\left({\text{F}}^{\prime }\right)\right]\right)\right) \end{array}$$ (4)

where **F**′ represents the channel-refined feature map, where average and max pooling over the channel dimension are represented by $\text{Av}{\text{g}}_{c}\left({\text{F}}^{\prime }\right)$ and $\text{Ma}{\text{x}}_{c}\left({\text{F}}^{\prime }\right)$, respectively, and [·, ·] represents concatenation. A 7 × 7 convolution followed by a sigmoid function σ(·) generates the spatial attention map **M**_*s*_ which highlights important regions within the feature representation. The additional feature maps generated by the channel and spatial attention mechanisms were forwarded to the subsequent layers for refinement. This process enhances the contrast, highlights the polyp regions, and maintains the crucial anatomical structures in the final output image.

### 3.4 Convolutional dense attention network (CDAN)

The proposed method is based on CDAN (Shakibania et al., 2025), which is used as a backbone model to enhance the illumination of colonoscopy images. The CDAN merges convolutional feature extraction and attention operations to upgrade the feature representation and emphasize the most important regions for diagnosis. The proposed CDMSP model includes a CDAN block consisting of a 3 × 3 convolution, ReLU activation, and CBAM attention module. This enables the network to extract features and enhance attention efficiently. The resulting feature map of the CDAN block is given by Equation 5.


$$\begin{array}{l} {\text{F}}_{\text{CDAN}}=\text{CBAM}(\sigma \left(\text{Conv}3\times 3\left(\text{F}\right)\right)) \end{array}$$ (5)

where **F**_CDAN_ represents the feature map produced by the CDAN, where in a 3 × 3 convolutional layer captures local spatial features from the input **F** succeeded by a nonlinear activation function σ(·). The features are enhanced by the CBAM attention module, which uses channel and spatial attention to highlight distinguishing anatomical elements, including mucosal texture and polyp borders. The enhanced feature maps of **F**_CDAN_ feature maps are later sent to the further layers for refinement and reconstruction, which helps the final image have better contrast, illumination correction, and visibility of clinically important structures, such as polyps.

### 3.5 Multi-scale pooling (MSP)

The proposed model incorporates an MSP layer to preserve local contrast and improve global lighting by gathering contextual information at different spatial scales. Given an input feature map *F*∈ℝ^*C*×*H*×*W*^, the MSP operation first performs channel reduction to obtain a compact feature representation for subsequent multi-scale processing. The channel-reduced feature map is given by Equation 6.


$$\begin{array}{l} {\text{F}}_{b}=\text{Con}{\text{v}}_{1\times 1}\left(\text{F}\right) \end{array}$$ (6)

where ${\text{F}}_{b}\in {\mathbb{R}}^{C^{\prime }\times H\times W}$ represents a feature map with a reduced number of channels obtained using a 1 × 1 convolution. This operation performs a channel-wise projection while maintaining the same time, spatial resolution, and reducing the computational cost. The MSP block first obtains the context information of the scene at different scales through average pooling using different kernel sizes. Then, the original resolution is produced by upsampling the image. The proposed MSP module captures contextual information through adaptive average pooling at scales 1 × 1, 2 × 2, and 4 × 4 scales. The features obtained from pooling are then upsampled using bilinear interpolation, concatenated, and integrated through a 1 × 1 convolution. The multiscale pooled feature maps are generated using (Equation 7).


$$\begin{array}{l} {\text{P}}_{k}=\text{Upsample}(\text{AvgPoo}{\text{l}}_{k}\left({\text{F}}_{b}\right)),\;k\in \left\{1,2,4\right\} \end{array}$$ (7)

where ${\text{P}}_{k}\in {\mathbb{R}}^{C^{\prime }\times H\times W}$ represents the multi-scale pooled feature map at scale (1, 2, 4) in which AvgPool_*k*_(·) extracts the contextual features, while Upsample(·) provides the same spatial resolution (*H*×*W*) after reintroducing the features. **F**_*b*_ is the channel-reduced map from the previous stage, and the final MSP representation is a multiscale pooled feature combination. The combined feature representation of the MSP module is given by Equation 8.


$$\begin{array}{l} {\text{F}}_{\text{MSP}}=\text{Con}{\text{v}}_{1\times 1}(\left[{\text{P}}_{1},{\text{P}}_{2},{\text{P}}_{4}\right]) \end{array}$$ (8)

The output feature map of the MSP module, indicated by ${\text{F}}_{\text{MSP}}\in {\mathbb{R}}^{C^{\prime \prime }\times H\times W}$ is a combination of multiscale features that are concatenated along the channel dimension and fused by a 1 × 1 convolution. From the Pyramid Pooling Module (PPM) in the Pyramid Scene Parsing Network (PSPNet) (Zhao et al., 2017), the new MSP layer pulls together context across multiple scales, boosting both local detail and overall scene understanding. For colonoscopy images, the exact magnification of both small and large anatomical features plays a key role in diagnosis, as abnormalities such as polyps can appear at very different scales. The combined multiscale features are then passed on to the reconstruction layers, which help achieve a higher level of lighting uniformity and enhance the clarity of the anatomical structures in the final output image.

### 3.6 Residual learning

Residual learning helps to stabilize network training by focusing on the difference between the input and desired output instead of directly predicting the target result (He et al., 2016). The network creates a residual map that shows the changes in illumination. This map was then added to the original image to generate an improved image. The predicted residual image is added to the input image, and a clipping operation is performed to limit the final output intensity within the range [0,1]. No activation function was added after the last 1 × 1 convolution. However, the residual output was combined with the input image, and the final pixel values were restricted to the interval [0,1]. This approach avoids the direct computation of full output transformation. For an input image *I*∈ℝ^*H*×*W*×3^, the output enhanced image is obtained by adding the learned residual map to the image. The enhanced output image is given by Equation 9.


$$\begin{array}{l} Î=\text{clip}\left(I+F\left(I\right),0,1\right) \end{array}$$ (9)

where *I*∈ℝ^*H*×*W*×3^ is the input RGB image, Î is the enhanced output, and *F*(·) is the residual mapping obtained by the network, where *I*+*F*(*I*) forms an improved image. The MSP unit gathers multi-scale features for computing F(I), which are subsequently mapped to the RGB residual space via a 1 × 1 convolution. The final enhanced image generated by the MSP features is given by Equation 10.


$$\begin{array}{l} Î=\text{clip}(I+\text{Con}{\text{v}}_{1\times 1}\left({\text{F}}_{\text{MSP}}\right),0,1). \end{array}$$ (10)

Where Î stands for the augmented output image, *I*∈ℝ^*H*×*W*×3^ indicates the input RGB image, and ${\text{F}}_{\text{MSP}}\in {\mathbb{R}}^{C\times H\times W}$ is the feature map obtained from the MSP module. A 1 × 1 convolution maps these features into the RGB residual space, the clip ( 0, 1) function limits the pixel values, and residual learning enhances the lighting and contrast. This technique improves brightness and contrast at the same time it keeps important anatomical features such as the edges of polyps, mucosal details and vascular patterns intact. These features are vital for accurate diagnosis based on colonoscopy image analysis.

### 3.7 Overall function of the network

Below is a detailed step-by-step elaboration of the enhancement method of the proposed model. The proposed CDMSP is a sequential enhancement pipeline model. An initial 3 × 3 convolution extracts low-level feature representations from the input RGB image. First, the network takes the input as RGB colonoscopy images with a size of 256 × 256 × 3 and generates enhanced RGB images while keeping the spatial resolution unchanged. The extracted features are further refined by two dense blocks, each with a 1 × 1 compression layer, followed by a CDAN block. The refined features are fed into the MSP module to capture the contextual features over various local regions of the image. Finally, a single layer of 1 × 1 convolution produces a residual image, which is then superimposed on the input to yield an enhanced image. The enhanced image is given by Equation 11.


$$\begin{array}{l} \hat{\text{I}}=\text{I}+f_{\theta }\left(\text{I}\right) \end{array}$$ (11)

where $\hat{\text{I}}$ is the enhanced image and **I** is the input image. The network, parameterized by the function *f*_θ_(**I**), generates a residual enhancement mapping that includes changes in illumination and contrast and retains the main structure. This formulation allows for effective image augmentation while maintaining the original visual content. Maintaining minute anatomical details, such as polyp edges, mucosal texture, and vascular patterns, is of paramount significance for accurate diagnosis from colonoscopy images.

## 4 Experimental setup

### 4.1 Dataset description

In this study, we used a modified CVC-ColonDB dataset version from the Kaggle. It contains 2,000 augmented colonoscopy images with corresponding annotation masks (Bernal et al., 2012). As there were no low-light image pairings available, we used a synthetic degradation model to generate low-light and reference image pairs for each reference image by simulating various realistic endoscopic degradations (brightness reduction, vignetting, Gaussian noise and blur, and minor color shifts). If the images were normal-light colonoscopy images, they became ground-truth images, whereas in the case of synthetically degraded images, they were only used as inputs to the network. Metrics based on full-reference that are listed in Tables 1 and 2 (SSIM, LPIPS, CFI, and EBCM) were calculated by contrasting the results of enhancement with their normal-light original ground truth images.

**Table 1:** Comparison of the proposed CDMSP model with state-of-the-art low-light enhancement methods on the CVC-ColonDB dataset.

Model	SSIM (↑)	LPIPS (↓)	EBCM (↑)	CFI (↑)	Time (ms/img) (↓)	FPS (↑)
RetinexNet Wei et al. (2018)	0.6814 ± 0.0919	0.1993 ± 0.0404	0.4942 ± 0.0664	87.4542 ± 3.4487	**20.1**	**49.86**
SWAMNET He et al. (2023)	0.6366 ± 0.0880	0.1615 ± 0.0301	0.5044 ± 0.0659	78.6254 ± 4.3167	55.0	18.17
RetinexFormer Cai et al. (2023)	0.6990 ± 0.0646	0.1879 ± 0.0329	0.4934 ± 0.0654	79.3305 ± 3.5586	35.5	28.15
SGLLIE Dong et al. (2025)	0.7253 ± 0.0647	0.1727 ± 0.0301	0.4394 ± 0.0517	82.7465 ± 3.1288	57.0	17.54
**CDMSP (Proposed)**	**0.8757** **±** **0.0408**	**0.0742** **±** **0.0243**	**0.5601** **±** **0.0691**	**95.1760** **±** **1.3383**	23.3	42.99

The best results in each column are bolded.

**Table 2:** Ablation study of the proposed CDMSP architecture on the CVC-ColonDB dataset.

Model	SSIM (↑)	LPIPS (↓)	EBCM (↑)	CFI (↑)
CDAN	0.8670 ± 0.0480	0.0641 ± 0.0234	0.5221 ± 0.0749	88.4606 ± 2.9952
CDAN–Dense	0.8708 ± 0.0483	0.0624 ± 0.0229	0.5256 ± 0.0762	88.6276 ± 3.0434
CDAN–MSP	0.8732 ± 0.0463	**0.0614** **±** **0.0219**	0.5277 ± 0.0747	88.7503 ± 2.9389
**CDMSP (Proposed)**	**0.8757** **±** **0.0408**	0.0742 ± 0.0243	**0.5601** **±** **0.0691**	**95.1760** **±** **1.3383**

The best results in each column are highlighted in bold.

There were no human-annotated normal-light ground-truth images of a clinical nature used in this quantitative assessment. The dataset was randomly split into different subsets for training (70%, 1400 image pairs), validation (10%, 200 image pairs), and testing (20%, 400 image pairs). A random seed of 42 was fixed during the dataset partitioning process to guarantee the reproducibility of splits in the experiments. Because no information identifying patients or source video clips was present in the downloaded dataset, it became impossible to make splits at the patient/video level, and the only feasible option was to split at the image level. Approximately 256 × 256 pixels was the uniform size of the training images after the initial resizing. Data augmentation on the fly was only enabled for the training data and should not be interpreted as adding more independent samples to the dataset.

### 4.2 Low-Light image simulation

A colonoscopy degradation pipeline was used to generate realistic low-light images from high-quality colonoscopy images for the training. It simulates non-uniform lighting, global brightness reduction, optical blur, sensor noise, and illumination-dependent color variation, which are characteristics of real colonoscopy imaging.

#### 4.2.1 Non uniform illumination

In colonoscopy imaging, uneven illumination is a result of the single source of light at the camera tip, which causes the centers to appear bright and the edges to appear dark. Therefore, a vignetting-based attenuation model was used to reproduce the effects.

#### 4.2.2 Global brightness reduction

Specifically, a global brightness reduction was employed to simulate the lower exposure caused by limited lighting and light absorption in the colon, thereby reducing the visibility of the colonoscopy images.

#### 4.2.3 Optical blur

Optical blur has been used to mimic various lens aberration effects and the movement of a camera, which can lead to the production of a series of blurred colonoscopy images during acquisition.

#### 4.2.4 Sensor noise

Sensor noise was introduced to simulate the phenomenon of noise amplification in low-light conditions, where a camera sensor increases the gain to enhance the signal, often leading to worsening of noise distortions.

#### 4.2.5 Illumination dependent color variation

Subtle changes made to specific color channels were used to simulate illumination-dependent color changes arising from tissue reflectance, changes in lighting, and camera white balance adjustment during colonoscopy acquisition. The proposed degradation pipeline simulates original low-light colonoscopy images in a sequence of non-uniform illumination, global brightness attenuation, optical blur, sensor noise, and color variations to create realistic images. Hence, it is a method for obtaining paired low-light and ground-truth images for the supervision of low-light image enhancement, as shown below. The degradation process is modeled by Equation 12.


$$\begin{array}{c} I_{\text{low}}=C\left(N\left(B\left(V\left(I_{\text{gt}}\right)\right)\right)\right) \end{array}$$ (12)

In this case, the term *I*_low_ refers to the artificially generated low-light photo, whereas *I*_gt_ denotes the ground truth image captured under normal lighting conditions. The symbols *V*(·), *B*(·), *N*(·), and *C*(·) denote vignetting, brightness reduction, camera noise/blur, and color alterations, respectively, which depend on illumination.

The degradation pipeline used a vignetting strength of 0.10 that was randomly sampled to give the exponent [1.0, 1.2] and the amount of fading was limited to be between [0.7, 1.0]. The intensity of the light (brightness) was also a factor for the simulation and was chosen with global scaling randomly within the range of [0.55, 0.80]; then a 3 × 3 Gaussian blur was applied with 0.1; finally, a zero-mean Gaussian noise at a noise level randomly sampled from [0.0008, 0.0015] was superimposed. Illumination-independent color shifts were accomplished through independent scaling of the three color channels (red, green, and blue) within the tight interval of [0.98, 1.02]. At the end of the pipeline, both degraded and target images were resized to 256 × 256 pixels.

### 4.3 Data preprocessing and augmentation

During training, paired geometric augmentations were synchronously applied to both the low-light input image and the corresponding ground-truth image to preserve pixel-wise correspondence. Specifically, random horizontal flipping, random vertical flipping, and random rotation ±15° were applied to each image. Photometric augmentations, including gamma correction (γ∈[0.8, 1.2]]), color jitter (brightness = 0.1, contrast = 0.1, saturation = 0.1), and Gaussian noise (standard deviation = 0.02), were applied only to the low-light input image to simulate realistic illumination variations while preserving original images. All images were resized to 256 × 256 pixels and converted to tensors before training. During validation and testing, only resizing to 256 × 256 pixels and tensor conversion were performed without random augmentations.

### 4.4 Hardware requirements and software specifications

The experiments were carried out on the Google Cloud Platform Colab by utilizing an NVIDIA Tesla T4 GPU (15 gigabytes (GB) Video Random Access Memory (VRAM)) and an Intel Xeon Central Processing Unit (CPU) along with 12 GB Random Access Memory (RAM). The remainder of the system included Ubuntu 20.04.6 Long-Term Support (LTS), CUDA 12.4, and NVIDIA driver 550.54.15. The proposed model was run on PyTorch with TorchVision support, and the full setup details are presented in Table 3.

**Table 3:** Hardware and software configurations for model training and evaluation.

Category	Specification
GPU	NVIDIA Tesla T4, 15 GB VRAM
CPU	Intel^®^ Xeon^®^, 2.00 GHz (2 cores, 4 threads)
RAM	≈ 12 GB
Architecture	x86_64
Operating System	Ubuntu 20.04.6 LTS
CUDA Version	12.4
NVIDIA Driver	550.54.15
Programming Language	Python 3.x
Libraries	PyTorch, Torchvision
Environment	Google Colab

### 4.5 Training configuration

#### 4.5.1 Optimization strategy

The CDMSP model was trained end-to-end using the Adaptive Moment Estimation (Adam) optimizer (Kingma and Ba, 2015), starting with a learning rate of 5 × 10^−5^ and employing a weight decay of 1 × 10^−5^ to assist the optimization in a stable manner and prevent overfitting. All the colonoscopy images 574 × 500 were resampled to 256 × 256 to be consistent with the inputs to the network. This setup ensured that the low-light image enhancement was properly trained. The learning rate schedule used was Cosine Annealing with Warm Restarts (CAWR) (Loshchilov and Hutter, 2017). The initial learning rate was set to 5 × 10^−5^. It was specified that *T*_0_ = 10 and *T*_mult_ = 2 were based on the desire to improve the optimization performance and avoid getting stuck in bad local minima. The model was trained for 100 epochs, with a batch size of 4. To prevent overfitting, an early stopping mechanism was implemented with a patience of ten epochs. The complete training setup is listed in Table 4.

**Table 4:** Training parameters used for the model optimization.

Parameter	Value
Original resolution	574 × 500
Image resize	256 × 256
Weight decay	1 × 10^−5^
Learning rate	5 × 10^−5^
Optimizer	Adam
Learning rate scheduler	CAWR
Epochs	100
Early stopping patience	10

#### 4.5.2 Loss function design

To maintain the structural details, visual quality, edge definition, and color accuracy in the enhanced colonoscopy images, a hybrid loss function was formulated using multiple objective functions. The entire optimization aim, represented as *L*_*total*_, leads to training the proposed network. The overall optimization objective used for network training is given by Equation 13.


$$\begin{array}{c} L_{total}=\lambda_{1}L_{SSIM}+\lambda_{2}L_{LPIPS}+\lambda_{3}L_{EBCM}+\lambda_{4}L_{CFI} \end{array}$$ (13)

where the overall optimization goal, referred to as *L*_*total*_ is the driving force behind the training of the proposed network. The loss terms *L*_*SSIM*_, *L*_*LPIPS*_, *L*_*EBCM*_, *L*_*CFI*_ represent losses in structural similarity, perceptual similarity, edge-based contrast, and color fidelity, consecutively. The scaling factors λ_1_, λ_2_, λ_3_, and λ_4_ are the adjustment coefficients that determine the extent to which each loss component affects the final output.

The SSIM (Wang et al., 2004) estimates the similarity between the enhanced and ground truth images in terms of luminance, contrast, and structural characteristics. Compared to pixel-wise losses, such as Mean Squared Error (MSE), SSIM aligns better with how humans visually perceive. SSIM loss is widely implemented to ensure the retention of structural features during image restoration. The SSIM loss is given by Equation 14.


$$\begin{array}{c} L_{SSIM}=1-SSIM\left(I_{pred},I_{gt}\right) \end{array}$$ (14)

Where *L*_*SSIM*_ denotes the structural similarity loss. *I*_*pred*_ and *I*_*gt*_ represent the predicted enhanced and ground truth images, respectively. The structural similarity between the two images is computed using the function *SSIM*(·). Subtracting 1 converts the similarity measure into a minimization objective. This loss term helps preserve anatomical structures, maintain mucosal texture patterns, and reduce structural distortions.

LPIPS measures perceptual similarity using deep features extracted from a pretrained VGG network (Zhang et al., 2018). Unlike pixel-based similarity measures, it covers semantic differences at a higher level, which is more consistent with human visual perception. The use of LPIPS loss in medical image enhancement helps preserve genuine textures, smooth color transitions, and complex structures that are essential for obtaining images. The LPIPS loss is given by Equation 15.


$$\begin{array}{c} L_{LPIPS}=LPIPS\left(I_{pred},I_{gt}\right) \end{array}$$ (15)

where *L*_*LPIPS*_ denotes the perceptual loss term. *I*_*pred*_ is the predicted (enhanced) image produced by the network, and *I*_*gt*_ is the corresponding ground-truth image. The operator *LPIPS*(·) calculates the LPIPS between two images in deep feature space. This loss is essential for maintaining realistic textures, ensuring that the color variations are natural, and reducing smoothing artifacts that are too strong. One of the key reasons for using colonoscopy image augmentation is the preservation of fine anatomical features such as the surfaces of polyps and mucosal patterns. This results in improved visual quality and more accurate clinical diagnosis.

Edge sharpness is a critical feature of colonoscopic images for accurate polyp and mucosal boundary identification. The EBCM loss was used to retain the edge information. First, the RGB images were converted to the luminance space. Next, Sobel operators were applied to obtain edge gradients. The edge-based contrast measure (EBCM) loss is given by Equation 16.


$$\begin{array}{c} L_{EBCM}=|E\left(I_{pred}\right)-E\left(I_{gt}\right)| \end{array}$$ (16)

where *L*_*EBCM*_ denotes the edge-based loss. *I*_*pred*_ and *I*_*gt*_ are the predicted image after enhancement and the ground-truth, respectively, while *E*(·) is a function that measures the mean edge strength, possibly through gradient operators such as Sobel filters. The direct subtraction of the edge intensities is a good way to maintain the intricate edge details and obtain better contrast in the dimmer areas of the images.

Color consistency is important in medical imaging because tissue color differences are often subtle and difficult to detect. The CFI loss quantifies the color discrepancies between the predicted and actual images in the CIE Lab color space. "The CFI loss is given by Equation 17


$$\begin{array}{c} L_{\text{CFI}}=|a_{\text{pred}}-a_{\text{gt}}|+|b_{\text{pred}}-b_{\text{gt}}| \end{array}$$ (17)

Where *L*_CFI_ is the color fidelity loss, *a*_pred_ and *b*_pred_ are the chromatic components of the enhanced image in the Lab color space, and *a*_*gt*_ and *b*_*gt*_ are those of the ground-truth image. The differences in the absolute values between these components indicate chromatic deviations and lead the network to maintain color consistency during enhancement. This loss function aids in reducing color distortion and retaining clinically relevant color features in colonoscopy images. The loss weights were determined experimentally to ensure a trade-off between the structural and perceptual qualities. The values were fixed at λ_*SSIM*_ = 0.30, λ_*LPIPS*_ = 0.30, λ_*EBCM*_ = 0.20, and λ_*CFI*_ = 0.20. More importance was given to SSIM and LPIPS to focus on structural and perceptual fidelity, whereas edge and color preservation received moderate weights so that the model did not produce overenhancement artifacts. This balanced training allows the model to brighten the scene while maintaining edges and producing perceptually reliable results.

### 4.6 Evaluation metrics

Four quantitative metrics were selected to evaluate the proposed CDMSP method: SSIM, LPIPS, EBCM, and CFI. These metrics measure structural similarity, perceptual quality, edge preservation, and color fidelity.

#### 4.6.1 SSIM

The SSIM is a method for assessing the structural similarity between two images by comparing their luminance, contrast, and structural details (Wang et al., 2004). Instead of operating on individual pixels, the SSIM measures the similarity of structures, which is more consistent with the human visual perception. The structural similarity index (SSIM) between two images is given by Equation 18.


$$\begin{array}{c} \text{SSIM}\left(x,y\right)=\frac{\left(2\mu_{x}\mu_{y}+C_{1}\right)\left(2\sigma_{xy}+C_{2}\right)}{\left(\mu_{x}^{2}+\mu_{y}^{2}+C_{1}\right)\left(\sigma_{x}^{2}+\sigma_{y}^{2}+C_{2}\right)} \end{array}$$ (18)

where μ_*x*_ and μ_*y*_ represent the mean intensities of images and, respectively, $\sigma_{x}^{2}$ and $\sigma_{y}^{2}$ are their variances, and σ_*xy*_ is the covariance between them. The constants *C*_1_ and *C*_2_ ensure the stability of the division. The SSIM varies from 0 to 1, and the closer the value is to 1, the better is the structural similarity. The SSIM is particularly important for colonoscopy image enhancement. It is primarily involved in preserving the anatomical characteristics of the images, such as polyp borders, mucosal textures, and vascular patterns. This, in turn, allows for accurate clinical interpretation and diagnosis of the disease in the future.

#### 4.6.2 LPIPS

LPIPS is a deep learning-based perceptual metric that computes image similarity using feature representations extracted from pretrained networks, such as VGG (Zhang et al., 2018). In contrast to pixel-wise metrics, LPIPS quantifies perceptual differences within a learned feature space that is well aligned with human visual perception. Lower LPIPS scores indicate greater perceptual similarity. LPIPS plays a vital role in colonoscopy image enhancement when evaluating whether the perceptual quality is preserved, including components such as real textures, uninterrupted color changes, and detailed anatomical features such as polyp surfaces and mucosal patterns. This ensures that the enhanced images are not only visually consistent but also clinically trustworthy for accurate diagnosis of the disease.

#### 4.6.3 EBCM

The preservation of structural edge information in intensified colonoscopy images using the EBCM method was examined (Palanisamy et al., 2020). This is because edge-based contrast measures are frequently applied in the assessment of structural image contrast and edge-conservation. First, both the reference and enhanced RGB images were transformed into grayscale images, and the horizontal and vertical image gradients were obtained using the Sobel operator (Kanopoulos et al., 1988). The gradient magnitude of each pixel is given by Equation 19


$$\begin{array}{ccc} G\left(i\right) & = & \sqrt{G_{x}{\left(i\right)}^{2}+G_{y}{\left(i\right)}^{2}}, \end{array}$$ (19)

$$\begin{array}{ccc} S\left(i\right) & = & \frac{\min\left(G_{r}\left(i\right),G_{e}\left(i\right)\right)}{\max\left(G_{r}\left(i\right),G_{e}\left(i\right)\right)} \end{array}$$ (20)

where *G*_*x*_(*i*) and *G*_*y*_(*i*) represent the horizontal and vertical Sobel gradients at pixel (i), respectively. For pixel (i), G(i) is the gradient magnitude, and S(i) is the edge similarity of pixel (i). Let *G*_*r*_(*i*) and *G*_*e*_(*i*) denote the gradient magnitudes of the reference and enhanced images, respectively. The Edge-Based Contrast Measure (EBCM) is given by Equation 21.


$$\begin{array}{c} EBCM=\frac{1}{N}\overset{N}{\underset{i=1}{\sum }}S\left(i\right) \end{array}$$ (21)

where *N* denotes the total number of pixels, and the EBCM ranges from 0 to 1, where a higher value indicates that the edge structures of the enhanced image closely match those of the original image. Edge information plays a crucial role in colonoscopy images because polyp boundaries, mucosal folds, and vascular patterns can be clearly identified by gradient changes. Therefore, EBCM can be a numerical indicator of the enhancement in images through stronger edges and better preservation of details in the structures.

#### 4.6.4 CFI

The CFI was used to assess the proximity of the colors of the enhanced image to those of the reference image. Initially, both images were transformed into the CIELAB (Lab^*^) color space, and their perceptual color differences were measured using the CIEDE2000 Δ*E*_00_ metric (Sharma et al., 2005). The Color Fidelity Index (CFI) is given by Equation 22.


$$\begin{array}{c} \text{CFI}=\max\left(0,100-\bar{\Delta E_{00}}\right) \end{array}$$ (22)

The average CIEDE2000 color difference was transformed into a Color Fidelity Index (CFI) as $\bar{\Delta E_{00}}$ is the mean CIEDE2000 color difference calculated for all the pixels involved. A color fidelity score (CFI) was applied, which only spans between 0 and 100. According to the results, a higher CFI indicates good color fidelity and a close resemblance to the reference. However, a lower value indicates greater color distortion. The CFI is a direct transformation of the CIEDE2000 color difference, which means that a smaller Δ*E*_00_ results in a higher CFI. Accurate color reproduction is crucial for colonoscopy images because even slight mucosal and vascular color changes can indicate pathological abnormalities. The CFI is a useful tool for quantitatively measuring the extent to which the colors remain the same in the enhanced images.

The evaluation metrics were carefully selected to assess the performance of the proposed CDMSP model. SSIM was used to evaluate the structural information similarity of the images, LPIPS was used to examine the perceptual level of the image, EBCM was used to assess edge preservation, and CFI was used to evaluate the color fidelity of the enhanced image. These four criteria work together to provide a comprehensive measure of structural preservation, perceptual quality, edge fidelity, and color fidelity.

### 4.7 Baseline and comparative models

To test the effectiveness of the proposed CDMSP model, a series of ablation and comparative experiments were performed on the CVC-ColonDB dataset. The CDAN was selected as the baseline model. To explore how dense connections and MSP contribute to improving feature representation, two modified versions, CDAN-Dense and CDAN-MSP, were created. In addition, the study also examined four modern top-performance low-light image enhancement techniques, namely RetinexNet (Wei et al., 2018), SWAMNet (He et al., 2023), RetinexFormer (Cai et al., 2023), and SGLLIE (Dong et al., 2025), which were used as points of comparison. All models were trained and tested using an identical split of the dataset to ensure a fair comparison. The numerical results were derived using SSIM, LPIPS, EBCM, and CFI, which stand for structural similarity, perceptual quality, edge preservation, and color fidelity, respectively.

## 5 Results and discussion

### 5.1 Quantitative evaluation

Table 1 presents the quantitative results for the various methods. The proposed CDMSP model outperformed both baseline variants and state-of-the-art methods across multiple evaluation metrics. In particular, higher SSIM and CFI values, lower LPIPS values, and better EBCM indicate stronger structural retention, higher perceptual quality, and improved edge sharpening and contrast in low-light colonoscopy images. RetinexNet, SWAMNet, RetinexFormer, and SGLLIE were selected as representative low-light enhancement methods for comparison with the proposed model. The latter model achieved the highest SSIM at 0.8757 ± 0.0408, which is significantly better than RetinexNet 0.6814 ± 0.0919, SWAMNet 0.6366 ± 0.0880, RetinexFormer 0.6990 ± 0.0646, and SGLLIE 0.7253 ± 0.0647 (Table 1). This improvement indicates that dense feature aggregation and MSP effectively preserve the structural features of colonoscopy images.

The proposed CDMSP model achieved the lowest LPIPS value of 0.0742 ± 0.0243 for perceptual similarity, which was far better than those of RetinexNet (0.1993 ± 0.0404), SWAMNet (0.1615 ± 0.0404), RetinexFormer (0.1819 ± 0.0329), and SGLLIE (0.1727 ± 0.0301) models. In addition, CDMSP obtained the highest EBCM score of 0.5601 ± 0.0691, indicating better edge retention and contrast enhancement than the other methods. The findings show that dense feature aggregation coupled with multiscale pooling substantially improves the perceptual quality and structural features of the colonoscopy images. The proposed CDMSP model achieved the highest CFI score of 95.1760 ± 1.3383 for color fidelity, which was significantly better than that of SWAMNet 78.6524 ± 4.3167 and other competing methods. In terms of computational efficiency, RetinexNet was the fastest, with an inference time of 20.1 ms per image, whereas CDMSP operated at 23.3 ms per image (42.99 FPS) and remained close to real-time. In contrast, transformer-based methods, such as RetinexFormer, require a much higher computational cost (35.5 ms per image). CDMSP strikes a better balance between quality improvement and computational efficiency than other methods.

**Figure 3:**
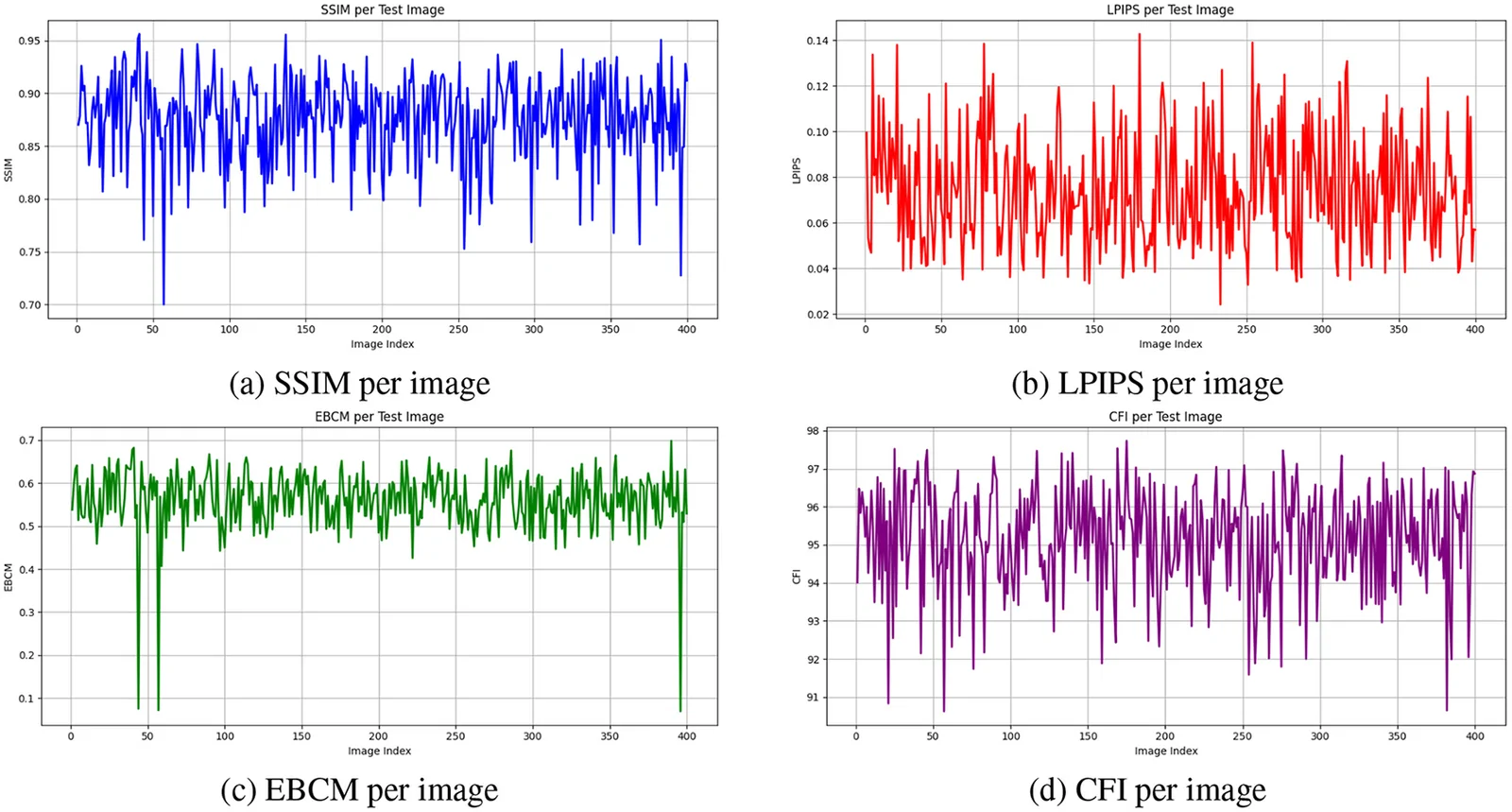
Per-image distribution of quantitative evaluation metrics on the CVC-ColonDB dataset (Bernal et al., 2012). The distributions indicate a consistent enhancement performance across diverse low-light colonoscopy samples. **(a)** SSIM (Wang et al., 2004), **(b)** LPIPS (Zhang et al., 2018), **(c)** EBCM (Palanisamy et al., 2020), and **(d)** CFI (Sharma et al., 2005).

Although the proposed CDMSP model clearly shows a marked improvement in performance over existing methods, it is still important to know how much each architectural component contributes. We conducted an ablation study to determine how dense feature aggregation and MSP separately influenced the performance. Table 1 shows the numerical comparison, and Figures 3a–d depicts the distribution of SSIM, LPIPS, EBCM, and CFI per image in the test set. As shown in Figure 3a, the SSIM scores were mostly stable across the samples, with the majority being above 0.85. Even though there were small changes at times in the most difficult low-light conditions, the SSIM values were always in line, which shows a reliable preservation of anatomical details.

Most of the LPIPS scores were near zero, indicating a very high visual similarity between the augmented and reference images, as shown in Figure 3b. The EBCM curves shown in Figure 3c support the idea that the proposed model not only retains edges but also effectively highlights the contrast. Most of the data points were clustered at the high end of the scale, whereas a few outliers were related to images captured under extremely dim-light conditions. The stable EBCM pattern revealed that the MSP technique effectively maintained detailed structural information. Likewise, the CFI diagram shows excellent color accuracy with very little fluctuation across the set, suggesting uniform enhancement and reliable color correction in low-light colonoscopy images.

The results of the ablation study, shown in Table 2, confirm the roles of dense feature aggregation and MSP. Both CDAN-Dense and CDAN-MSP not only enhanced the SSIM but also reduced the LPIPS of the baseline CDAN. The proposed CDMSP model produced the best results for SSIM (0.8757), EBCM (0.5601), and CFI (95.1760), indicating strong structural similarity, edge definition, and color consistency. However, its LPIPS score (0.0742) was slightly higher than those of the individual CDAN variants, implying that the integration of dense feature aggregation and MSP could result in feature-level changes that are captured by the LPIPS metric. Despite this trade-off, CDMSP has shown remarkably high levels of SSIM, EBCM, and CFI, which stand for a greater degree of structural similarity, better preservation of edges, and better matching of original colors when compared with the models subjected to ablation.

### 5.2 External dataset validation

To evaluate the performance of the CDMSP model on unseen low-light degradation data, we performed experiments not only on the primary dataset but also on another publicly available dataset of colonoscopy images, the Kvasir-SEG dataset (Jha et al., 2020). For a fair comparison, the synthetic low-light degradation protocol and evaluation procedure were kept the same as those in the main dataset. The results summarized in Table 5 show that the model achieved a mean SSIM of 0.8750 ± 0.0342, LPIPS of 0.1000 ± 0.0268, EBCM of 0.5555 ± 0.0669, and CFI of 94.6711 ± 1.3774 on CVC-ColonDB (Bernal et al., 2012).

**Table 5:** Performance of the proposed CDMSP model on two public colonoscopy datasets using the same synthetic low-light degradation protocol.

Dataset	SSIM ↑	LPIPS ↓	EBCM ↑	CFI ↑
CVC-ColonDB (Bernal et al., 2012)	0.8750 ± 0.0342	0.1000 ± 0.0268	0.5555 ± 0.0669	94.6711 ± 1.3774
Kvasir-SEG (Jha et al., 2020)	0.8472 ± 0.0370	0.1333 ± 0.0247	0.5457± 0.0434	94.8988 ± 0.9541

Values are reported as mean ± standard deviation. Higher SSIM, EBCM, and CFI values indicate better performance, whereas a lower LPIPS is preferred.

On the Kvasir-SEG dataset, which was not part of the training set, the model's performance was a mean SSIM of 0.8472 ± 0.0370, LPIPS of 0.1333 ± 0.0247, EBCM of 0.5457± 0.0434, and CFI of 94.8988 ± 0.9541. The encouraging results on these datasets indicate that the proposed CDMSP method can generalize well when given new colonoscopic images from a public dataset. This is likely because the model structure allows it to pick up various sources of features in different images.

### 5.3 Statistical analysis

The statistical significance of the differences in performance was tested by first performing a one-way Analysis of Variance (ANOVA) (Fisher, 1925), followed by Tukey's Honestly Significant Difference (HSD) (Tukey, 1953) analysis as a post-hoc test on the main test group data set. The ANOVA results are summarized in Table 6, which presents the one-way ANOVA results for the ablation study on the primary test dataset. Significant differences were observed among all evaluation metrics (SSIM, LPIPS, EBCM, and CFI; *p* < 0.05). Tukey HSD analysis further showed that CDMSP outperformed both the baseline method and the ablated models in a statistically significant manner, particularly for LPIPS, EBCM, and CFI. These findings highlight the statistical reliability of the proposed CDMSP method.

**Table 6:** One-way ANOVA results for the ablation study of the primary test dataset.

Metric	*F*	*p*-value	$\eta_{p}^{2}$
SSIM	3.088	0.0263	0.0058
LPIPS	27.884	< 0.001	0.0498
EBCM	6.792	< 0.001	0.0126
CFI	598.419	< 0.001	0.5294

A one-way ANOVA and Tukey's Honestly Significant Difference (HSD) post-hoc test were conducted to assess the statistical significance of the performance differences between the compared state-of-the-art methods using the primary test dataset. As shown in Table 7, statistically significant differences were observed for all assessment criteria, such as SSIM, LPIPS, EBCM, and CFI, with *p* < 0.001, and $\eta_{p}^{2}$ values fluctuated from 0.5454 to 0.7745. The Tukey HSD results also demonstrated that the proposed CDMSP model exhibited much better performance than the state-of-the-art methods in all aspects considered *p* < 0.05.

**Table 7:** One-way ANOVA results for the state-of-the-art comparison on the primary test dataset.

Metric	*F*	*p*-value	$\eta_{p}^{2}$
SSIM	635.134	< 0.001	0.5601
LPIPS	1548.594	< 0.001	0.7564
EBCM	598.334	< 0.001	0.5454
CFI	1713.418	< 0.001	0.7745

### 5.4 Convergence analysis

**Figure 4:**
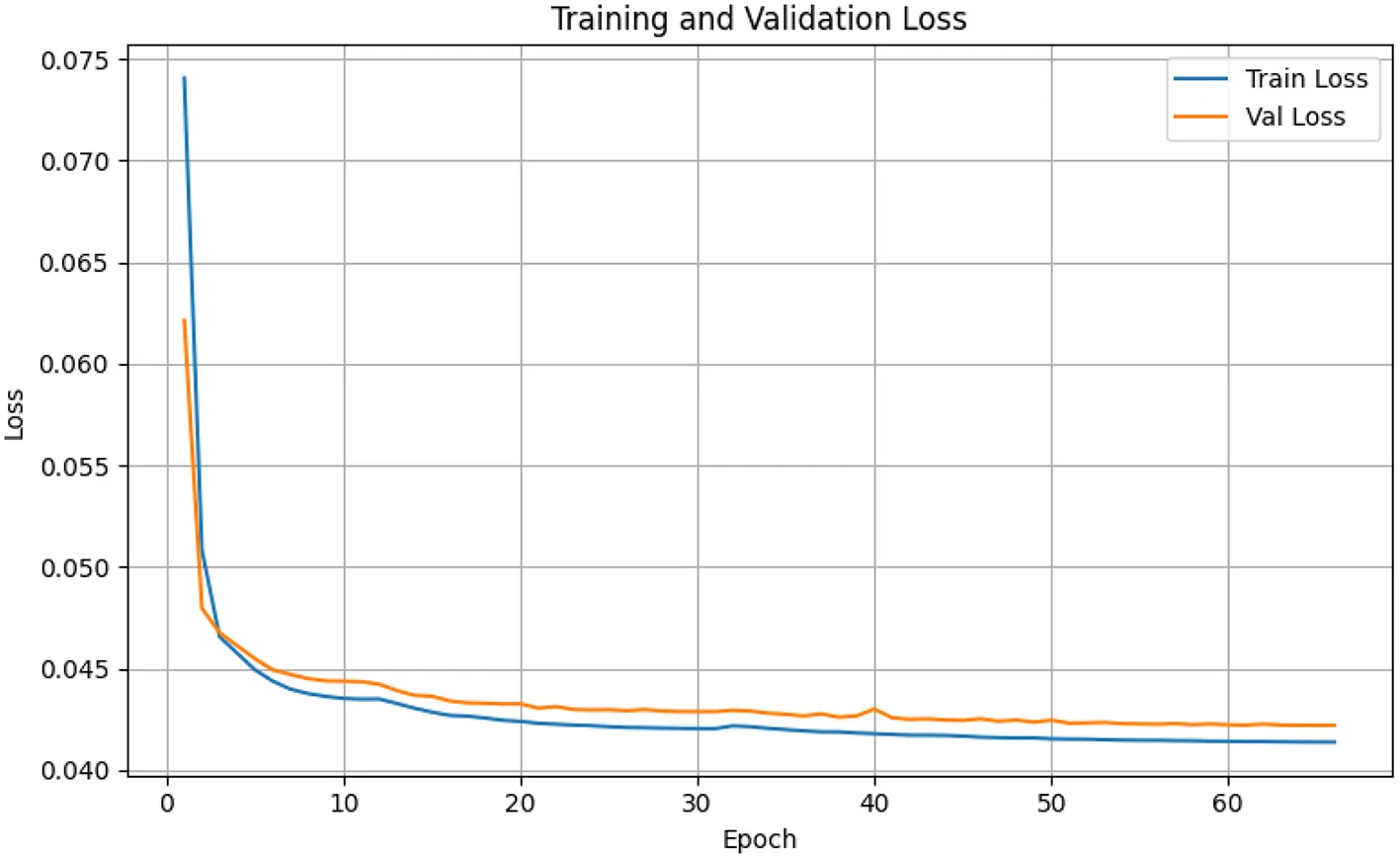
Training and validation loss curves of the proposed CDMSP model on the CVC-ColonDB dataset (Bernal et al., 2012). The curves demonstrate rapid initial convergence, followed by stable optimization with a minimal generalization gap.

Figure 4 shows that the CDMSP model gradually improved and updated its results at each training step. The number of errors or losses decreased rapidly at the beginning of the training for both the training and validation sets. It started at around 0. 074 and decreased below 0. 047 in only a few steps. After that, the curves got closer and closer to about 0. 041 without any noticeable increase or decrease, indicating that the model's optimization was quite steady. These two, the Adam optimizer and a learning rate scheduler called CAWR, help with this smooth convergence. The training and validation loss curves run almost parallel to each other throughout the optimization process, which means that we have an extremely small generalization gap and very little overfitting. The validation loss did not increase at any point in time. It continually decreased and became stable after approximately 30–40 epochs, indicating that the model had found a local minimum. Stopping training early if the validation loss does not improve for 10 epochs is a good strategy to avoid excessive training and reduce overfitting. The results show that the proposed CDMSP model was optimized in a consistent and efficient manner.

### 5.5 Qualitative evaluation

**Figure 5:**
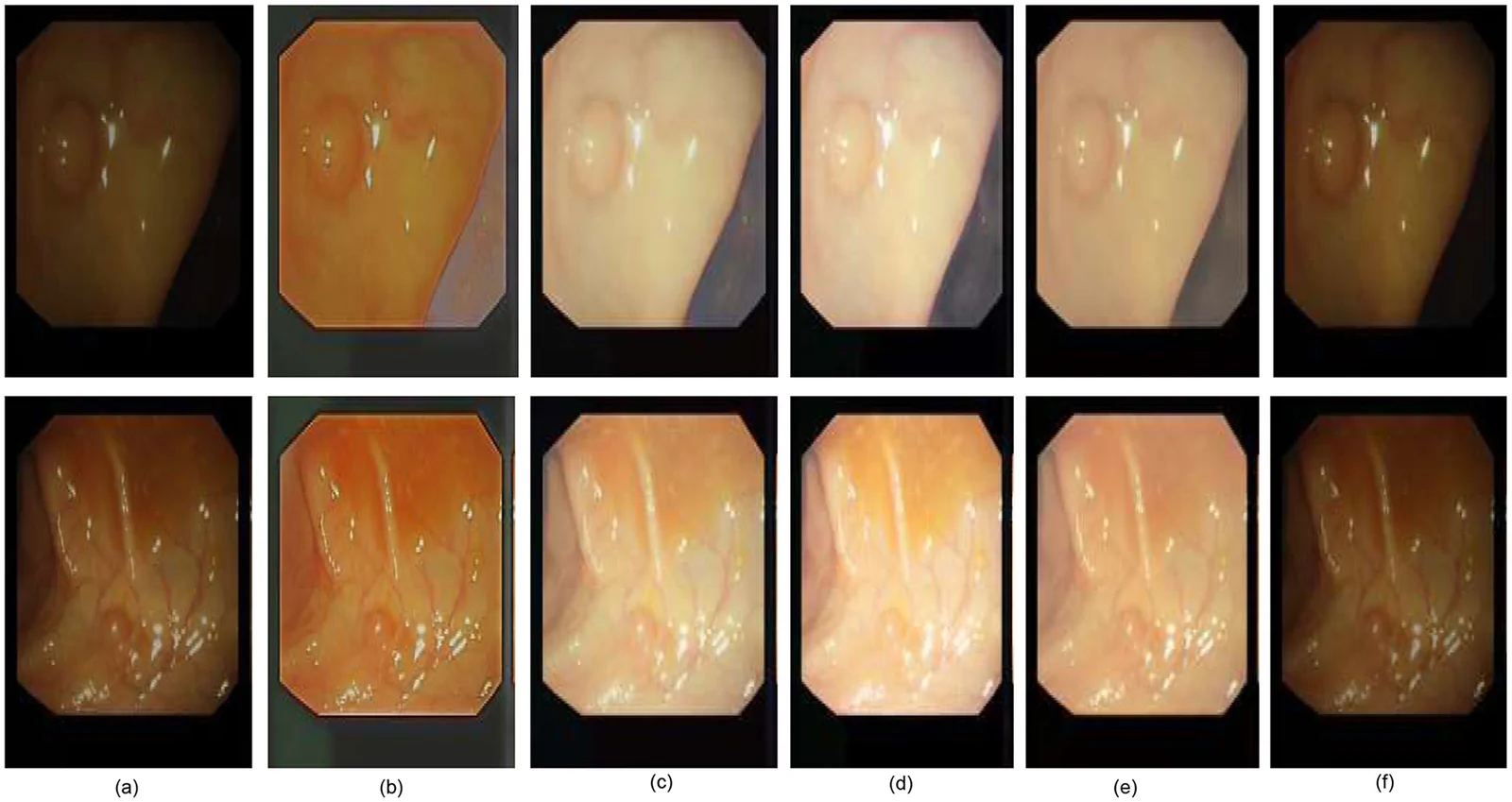
Qualitative comparison of low-light colonoscopy image enhancement methods. **(a)** Low-light input images from the CVC-ColonDB dataset (Bernal et al., 2012); **(b)** RetinexNet (Wei et al., 2018); **(c)** SwamNet (He et al., 2023); **(d)** RetinexFormer (Cai et al., 2023); **(e)** SGLLIE (Dong et al., 2025); and **(f)** the proposed CDMSP model. The proposed model achieves improved illumination, enhanced contrast, and better preservation of structural details compared with the competing methods.

Figure 5 presents a qualitative comparison of the proposed CDMSP model with other state-of-the-art methods, namely RetinexNet, SWAMNet, RetinexFormer, and SGLLIE. The first set of low-light colonoscopy images suffers from poor lighting, low contrast, and the absence of structural information. Although RetinexNet and SWAMNet are successful in increasing brightness, they also smooth excessively, distort colors, and make the contrast patchy. RetinexFormer brightens the image but results in overexposed areas that reduce the visibility of texture details, whereas SGLLIE improves the lighting but fails to sufficiently preserve vascular patterns and boundaries.

**Figure 6:**
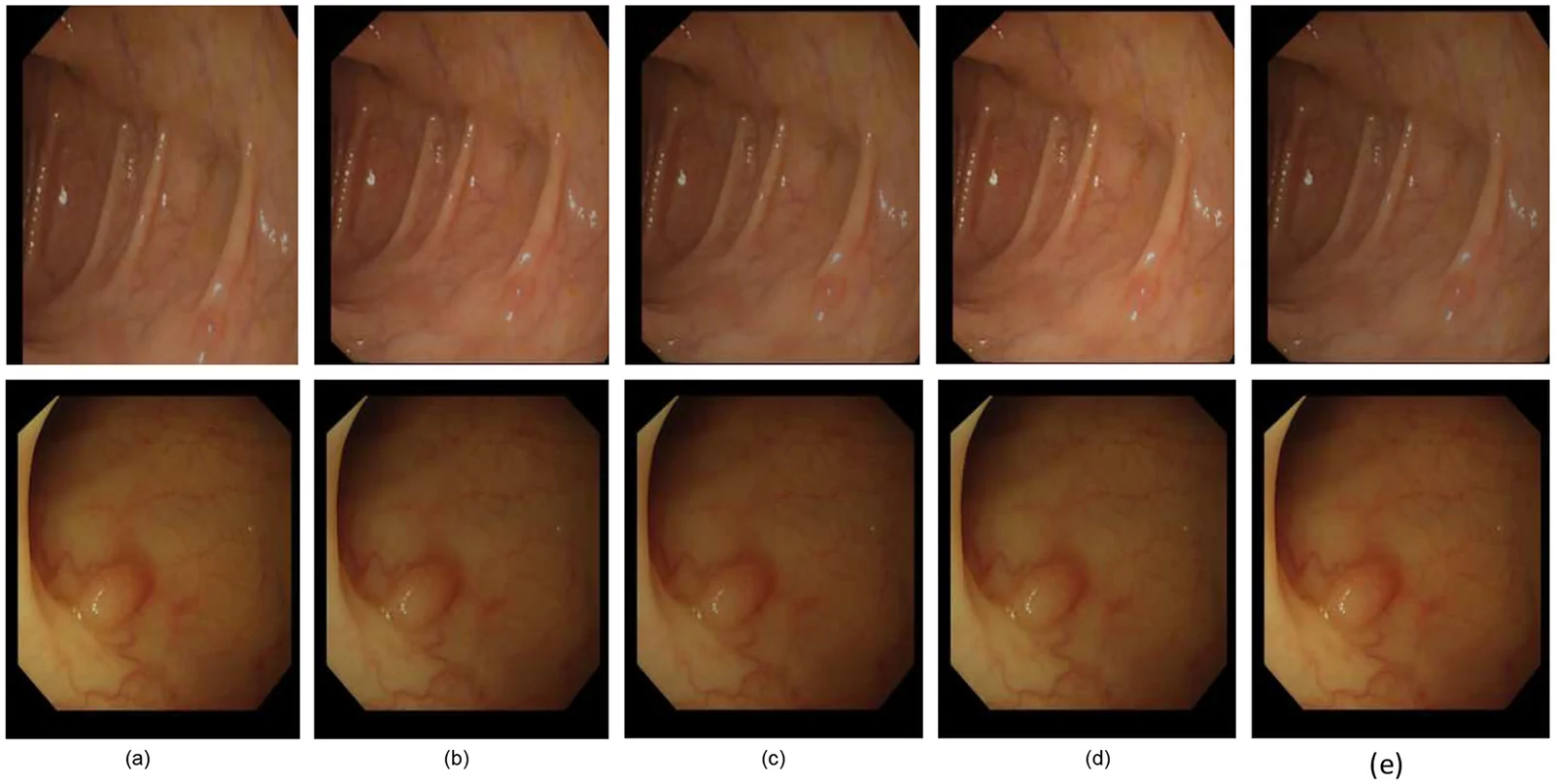
Qualitative ablation study of the proposed CDMSP model. **(a)** Low-light input images from the CVC-ColonDB dataset (Bernal et al., 2012), **(b)** CDAN, **(c)** CDAN-Dense, **(d)** CDAN-MSP, and **(e)** the proposed CDMSP model. The progressive integration of dense and MSP modules improves illumination, contrast, and structural detail preservation.

The proposed CDMSP model visually balances the enhancements with natural colors and improves the structural clarity. Compared with the original images, the enhanced images clearly show mucosal textures and vascular features without obvious artifacts or overexposure effects. The proposed CDMSP model can preserve the complex structural traits and subtle tissue patterns of colonoscopy images in a highly effective manner. This improvement is due to better feature extraction and attention mechanisms, which lead to results that are visually consistent with clinical readings. Figure 6 shows a qualitative ablation study comparing the CDAN, CDAN-Dense, CDAN-MSP, and proposed CDMSP methods.

The original low-light images (Figure 6a) are poorly illuminated, almost lack contrast, and the mucosal features are barely discernible, whereas the CDAN baseline (Figure 6b) improves brightness, but the brightness is uneven. Adding dense connections (Figure 6c) increases the spread of features and enhances the representation of local textures. The CDAN-Dense model provided better lighting and visibility of mucosal folds and vascular structures than the baseline model. For the CDAN-MSP variant, multi-scale contextual feature extraction was used for a better structural representation. Figure 6e shows that the latest CDMSP version provides the most balanced improvements, maintaining the structural features intact without causing color distortion or overenhancement.

### 5.6 Complexity analysis

The proposed model is a lightweight Fully Convolutional Network (FCN) with only 0.124 million trainable parameters. Debugging takes 23.3 ms per image, which is equivalent to 42.99 FPS. The results demonstrate the computational efficiency of the proposed method and its potential for real-time enhancement of low-light colonoscopy images in hospitals.

### 5.7 Failure case analysis

**Figure 7:**
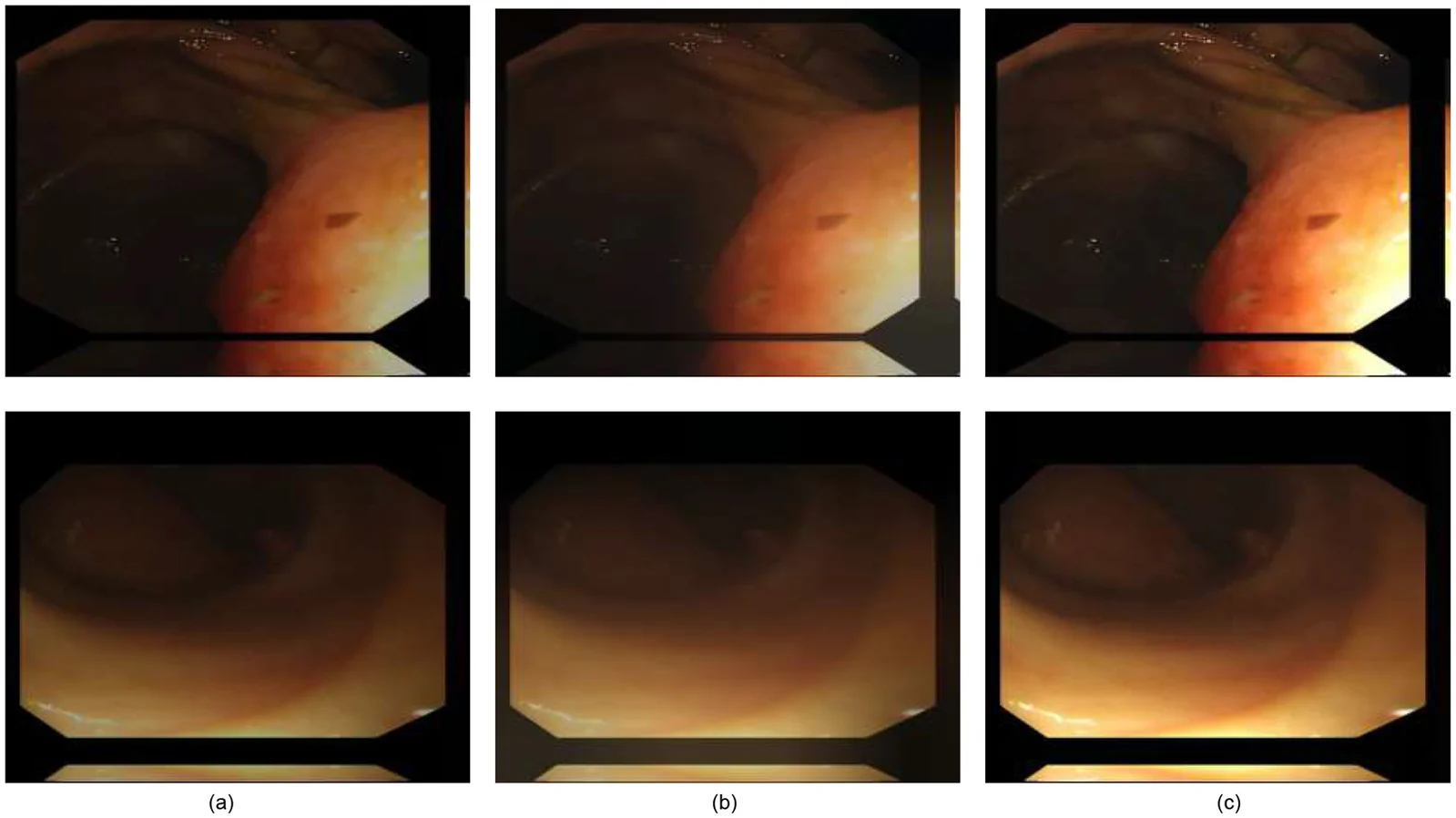
Failure case analysis of the proposed CDMSP model. **(a)** Low-light input, **(b)** enhanced output, and **(c)** ground-truth reference.

Although the proposed CDMSP model can provide considerable enhancement performance for nearly all test images, there is still a significant issue under extremely low-light conditions. As shown in Figure 7, extremely underexposed areas and non-uniform illumination make it difficult to retrieve fine anatomical details and may also result in slight variations in brightness. Therefore, future research should focus on enhancing the robustness of the model under the most extreme illumination conditions.

### 5.8 Limitations and future work

The first shortcoming of this study is that the downloaded Kaggle-distributed dataset did not contain patient- or video-level metadata; therefore, data partitioning at the patient or video level could not be performed. This implies that the dataset was split at the image level. Further research will test the proposed model on datasets with patient- or video-level annotations to reduce the risk of data leakage and offer a stronger evaluation of the model's ability to generalize. Each model was trained once by following a fixed experimental protocol with a fixed random seed, which was performed because of the high computational cost of training deep learning models. This approach not only ensured a fair comparison of the methods but also provided an opportunity for deeper exploration of the variability of the results, which could come from repeated training. This will be a part of our future research.

Ultimately, this study aimed to devise an enhancement system for low-light colonoscopy images that can be further used in downstream clinical tasks, while it will not directly focus on tasks such as polyp detection or segmentation. The clinical relevance of the improved images was not assessed using tasks such as polyp detection, segmentation, or blinded clinical assessment. The proposed enhancement network can be connected to the endoscopic image-processing pipeline as a real-time preprocessing module. It can be used to improve colonoscopy images under low-light conditions before they are visualized or used for downstream CAD tasks, such as detection and segmentation.

Future work will assess the proposed setup through detection, segmentation, and clinical examination of polyps, performed in a blinded manner. This further proves its diagnostic value. We tested the newly devised CDMSP approach on an external public colonoscopy dataset; however, only low-light images were artificially created when we used full-reference metrics that required pairs of normal-light ground truth images. There seems to be a lack of publicly accessible datasets that contain authentic low-light colonoscopy images and corresponding images. In the future, we intend to perform validation and use the proposed technique on real clinical low-light colonoscopy images to fully determine its clinical application.

## 6 Conclusion

This study introduces a deep learning network designed to improve images from colonoscopy performed in low-light conditions, called CDMSP, which is a combination of attention refinement, dense connectivity, MSP, and edge-guided optimization. Testing on the CVC-ColonDB dataset showed that this new network generates results that are better than those of the current leading techniques by SSIM, LPIPS, EBCM, and CFI measurements, which represent higher levels of structure retention, visual appeal, edge sharpness, and color accuracy. These findings attest to the success of this design in enhancing colonoscopy images under dim light. Future work will focus on validating the model with larger clinical datasets and deploying it in live colonoscopy.

## Data Availability

The original contributions presented in the study are included in the article/supplementary material, further inquiries can be directed to the corresponding authors.
